# Passive Joint Forces Are Tuned to Limb Use in Insects and Drive Movements without Motor Activity

**DOI:** 10.1016/j.cub.2013.06.024

**Published:** 2013-08-05

**Authors:** Jan M. Ache, Thomas Matheson

**Affiliations:** 1 University of Leicester, Department of Biology, University Road, Leicester LE1 7RH, UK; 2 Department of Animal Physiology, Zoological Institute, Biocenter, University of Cologne, Zülpicher Strasse 47b, 50674 Cologne, Germany

## Abstract

**Background:**

Limb movements are generally driven by active muscular contractions working with and against passive forces arising in muscles and other structures. In relatively heavy limbs, the effects of gravity and inertia predominate, whereas in lighter limbs, passive forces intrinsic to the limb are of greater consequence. The roles of passive forces generated by muscles and tendons are well understood, but there has been little recognition that forces originating within joints themselves may also be important, and less still that these joint forces may be adapted through evolution to complement active muscle forces acting at the same joint.

**Results:**

We examined the roles of passive joint forces in insect legs with different arrangements of antagonist muscles. We first show that passive forces modify actively generated movements of a joint across its working range, and that they can be sufficiently strong to generate completely passive movements that are faster than active movements observed in natural behaviors. We further demonstrate that some of these forces originate *within the joint itself*. In legs of different species adapted to different uses (walking, jumping), these passive joint forces complement the balance of strength of the antagonist muscles acting on the joint. We show that passive joint forces are stronger where they assist the weaker of two antagonist muscles.

**Conclusions:**

In limbs where the dictates of a key behavior produce asymmetry in muscle forces, passive *joint* forces can be coadapted to provide the balance needed for the effective generation of other behaviors.

## Highlights

• Limb movements are assisted by passive forces arising *within joints*
• Functional cyclic movements can be generated by a single motor neuron
• Joint forces are matched to the strength of antagonist muscles across species
• Passive joint properties can transfer force from a stronger to a weaker muscle

## Graphical Abstract

**Figure:**
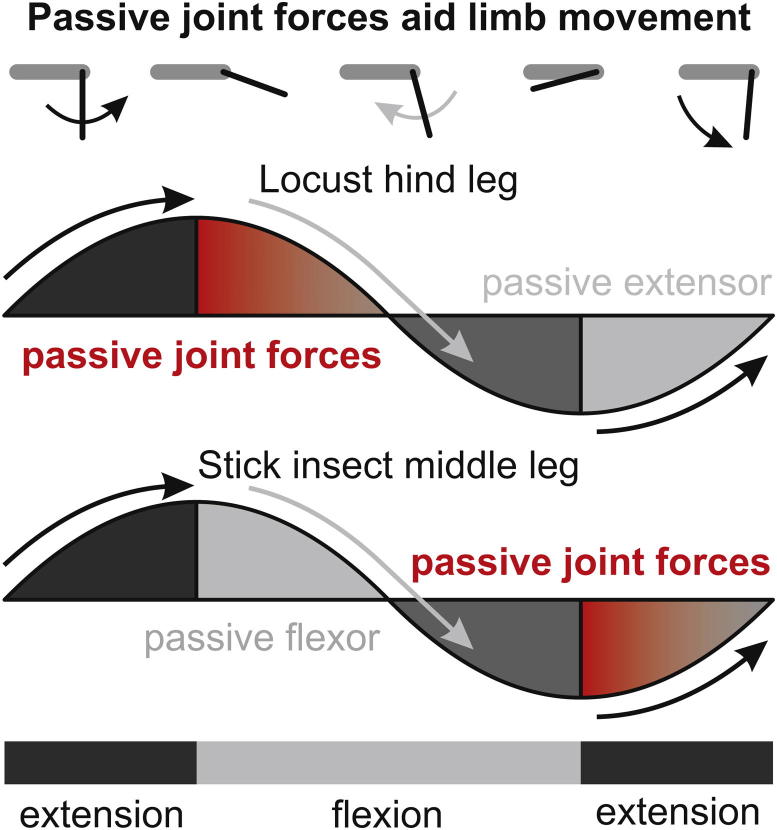

## Introduction

Most animal movements are driven by muscle contractions, but there is now a substantial body of evidence that passive forces, originating in muscles, tendons, or other tissues, interact with active forces to generate limb movements in both vertebrates and invertebrates [1–13]. We show that meaningful movements can be generated by forces arising within joints themselves. Restorative biomechanical forces can stabilize light limbs against the effects of gravity [7] and can act much more quickly than neuromuscular reflexes, which are constrained by relatively slow neuronal conduction velocities and muscular activation dynamics [11–13]. In some specialized limbs, storage of muscular energy by passive elastic structures [14, 15] permits the generation of ballistic movements that exceed maximal velocities of muscle shortening [16–18]. At another extreme, entirely passive bipedal robots (“passive-dynamic walkers”) can produce naturalistic gaits driven only by gravity [19] or by minimal active “muscle” force acting at a single joint [20]. In contrast to the extensive literatures on these subjects, passive forces originating in limb joints themselves (not in the muscles or their ligaments), particularly those acting during nonballistic movements, have been largely overlooked. Exceptions include the unguis (distalmost part of the foot) of most insects [1, 21–23] and the trochanter-femur (“hip”) joint of the cockroach [24]. In both of these, muscle contractions move the joint in one direction, but return movements arise passively.

Although passive forces in insect joints can be of the same order of magnitude as active forces [3, 7], it is not known whether they are specifically matched to active forces generated by muscles acting at the same joint or whether they are independent properties shaped by other selective pressures. We have previously shown that the resting angle of the locust hind leg femur-tibia (FT) joint is dominated by the extensor tibiae muscle, suggesting strong passive forces in the extensor tibiae muscle and weaker passive forces in the flexor tibiae muscle [8]. Here, we take a comparative approach to test the hypothesis that passive *joint* forces are matched to different muscle strengths at this joint, and to determine whether passive joint forces produce functionally relevant movements.

We compared the highly specialized [25] jumping legs of the locust *Schistocerca gregaria* (Orthoptera: Acrididae; see Figure S1A available online) to the unspecialized walking legs of the false stick insect *Pseudoproscopia scabra* (Orthoptera: Proscopiidae; Figure S1B), and to *Pseudoproscopia* hind legs, which reflect an intermediate condition. It is important to note, however, that our study did not examine jumping behavior. Instead, we focused on cyclical, nonballistic movements of the tibia made in the context of aimed scratching and the swing phase of walking and running, when the leg is unimpeded by ground contact [3, 5, 26–29].

Only two muscles, the extensor and flexor tibiae, act at the FT joint. In the locust, the extensor muscle is innervated by just two excitatory motor neurons, the fast (FETi) and slow (SETi) extensor tibiae, which can be reliably and independently stimulated in an isolated or denervated leg [8]. This provides an opportunity to systematically analyze the interactions between passive forces and active, muscle-driven forces in the complete absence of sensory feedback or conflicting motor signals. We have taken a direct and pragmatic approach to analyzing the importance of passive forces in generating unimpeded movements: we measured leg movements directly and made comparisons based on movement trajectories, amplitudes, and velocities. This avoids errors and assumptions that can be associated with inverse dynamics approaches. We generated natural active movements by stimulating FETi at spiking frequencies observed during natural behaviors. The movements we analyzed therefore reflect the interactions of active forces with passive assisting and resisting forces originating in both antagonist muscles and in the joint, acting through their normal lever arms. We examined legs with differing balances of strength between antagonist muscles to determine whether passive joint properties are balanced accordingly.

The locust hind leg extensor tibiae muscle is approximately 5 times heavier and 21 times stronger than the flexor tibiae muscle [25], which permits the generation of the large extension forces required for powerful jumps. In the stick insect *Carausius morosus*, which cannot jump, the flexor tibiae muscle of each leg is approximately 3 times stronger than the antagonistic extensor muscle [30], which reflects the demands of walking locomotion. *Pseudoproscopia*, which is more closely related to locusts than to stick insects, represents an intermediate situation. This insect can jump using its hind legs, but the extensor tibiae muscles of these legs are only 2.2 times heavier than the antagonistic flexor tibiae muscles. This is reversed in the middle (walking) legs, where the flexor tibiae muscles are 2.6 times heavier than their extensor antagonists.

We show that passive forces can generate rapid, functionally relevant movements. In the locust, we show that passive flexions of the hind leg tibia are driven by forces arising *within the joint itself*, not in the muscles or tendons; whereas extensions are driven by passive forces of the extensor tibiae muscle. This differs from the situation seen in the nonjumping stick insect *Carausius morosus*, where it is extensions that are driven by passive joint forces [7]. Analysis of *Pseudoproscopia* jumping and walking legs permitted us to demonstrate that in three species and four leg pairs in which antagonist muscles have different strengths, passive *joint* forces always support the weaker muscle. We therefore suggest that passive joint forces are shaped by evolutionary adaptation and form an important component of effective motor control.

## Results

### Cyclic Extension-Flexion Movements Can Be Driven by a Single Extensor Motor Neuron in the Absence of Active Flexor Muscle Contractions

**Figure 1:**
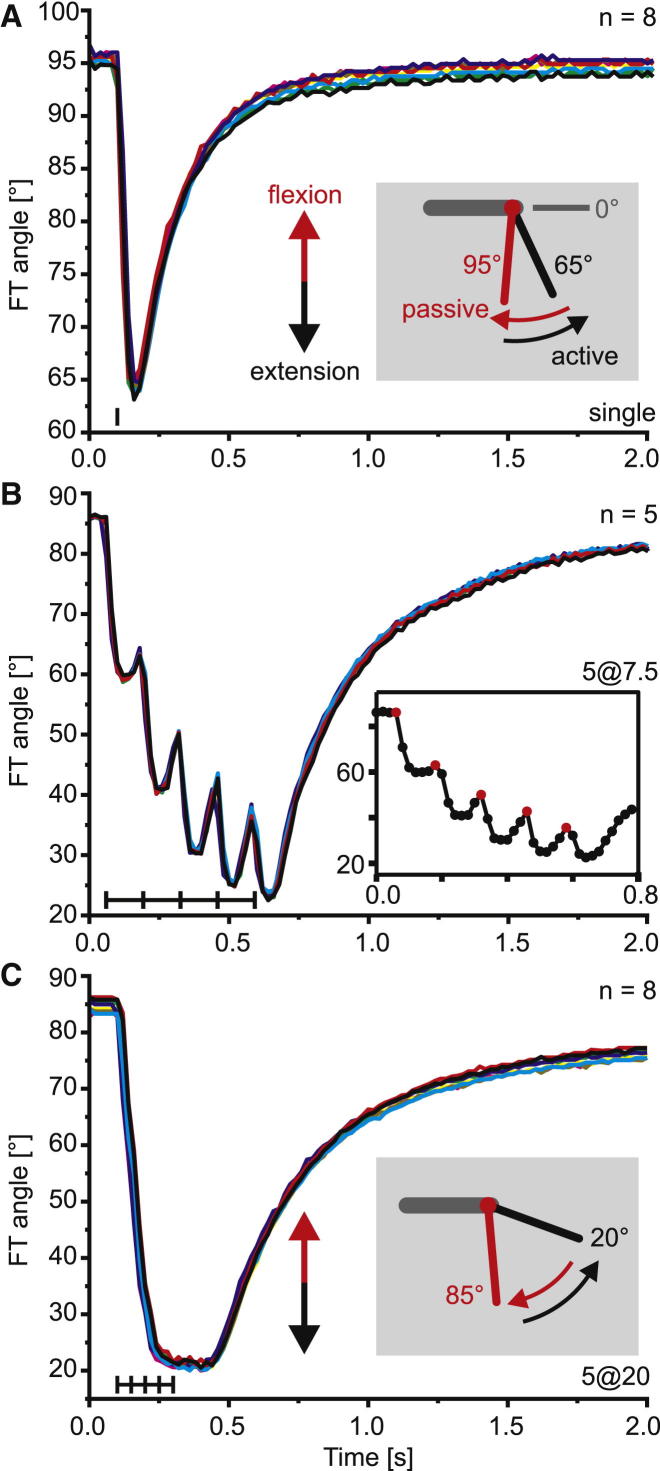
Activation of Just the Fast Extensor Tibiae Motor Neuron Generates Natural Extension and Subsequent Passive Flexion Movements in the Locust (A) Single fast extensor tibiae (FETi) motor neuron spikes elicited fast tibial extensions that did not carry the tibia to its fully extended angle of 19°. Each extension was followed by a passive flexion of the tibia toward the starting angle. The inset indicates the femorotibial joint angle notation (femur represented by gray bar, tibia represented by red bar [start position] and black bar [maximally extended position]) and directions of joint movement (arrows). (B) Five FETi spikes at 7.5 Hz elicited a series of extensions separated by smaller passive flexions. The inset shows at an expanded timescale the video sampling frequency in one example trial; each frame is represented by one dot, and the timing of the stimulus (to the nearest frame) is shown in red. (C) Five FETi spikes at 20 Hz elicited a full extension. The tibia remained fully extended for approximately 250 ms and then returned passively toward the starting angle. Each line represents a single trial of the same animal within each panel; each panel shows data from a different animal. Inset is as in (A). The black vertical tick marks in the lower left corner of each panel indicate the timing of stimulus pulses. Note that movement could begin between frames. All trials were aligned to stimulus onset. All movements started from the current resting state, i.e., the start angle was not imposed. FT, femur tibia. See also Table S1.

We elicited controlled patterns of spiking in FETi in denervated hind legs of seven otherwise intact locusts and measured the resulting movements (Figure 1). Repeated stimulus trains led to highly similar movements in every trial, regardless of whether we elicited single FETi spikes (Figure 1A), trains of FETi spikes at 7.5 Hz (Figure 1B), or trains at 20 Hz (Figure 1C). After each FETi-driven extension movement, the tibia returned toward its initial starting position. These return movements were entirely passive and not driven by active contractions of the flexor tibiae muscle, since the leg was denervated. Variability of these passive return movements was low (Figures 1A–1C). Increasing the number or frequency of FETi spikes had only a small effect on the peak velocity of active extension movements: single-pulse stimulation drove tibial movements at 810°/s, whereas stimulation with five pulses at 20 Hz led to an increase of only 6%, to 860°/s (Table S1). Single-pulse stimulation of FETi led to relatively small-amplitude active extensions that drove the tibia to an angle of about 65° (Figure 1A; Table S1). Stimulation of FETi with five pulses at 7.5 Hz led to larger-amplitude movements, driving the tibia to 25°, and stimulation with five pulses at 20 Hz led to the largest-amplitude movements, driving the tibia to 21° (Figure 1; Table S1).

When FETi was stimulated to spike five times at 7.5 Hz, each of the five FETi-driven extension twitches was followed by a passive return movement (Figure 1B). As a consequence, the tibia made a cyclic sequence of fast extension and flexion movements that closely resemble movements made during aimed scratching [5], despite the complete lack of flexor tibiae muscle contraction.

### Passive Forces Modulate Active Movements across the Working Range of the Joint

**Figure 2:**
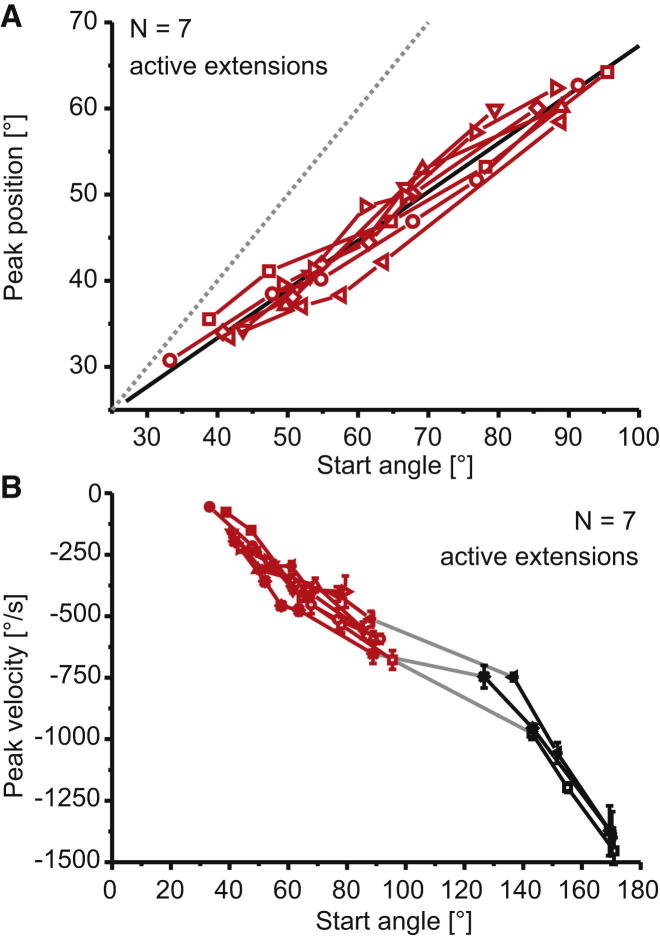
Passive Forces Shape Active, Muscle-Driven Extensions (A) The peak position reached by the tibia during active extensions elicited by a single FETi spike depended on the starting position. Each point is the mean peak amplitude of 5–9 extensions in one animal (N = 7, indicated by different symbols). Error bars were omitted for clarity, but the within-animal variability was very low (see Figure S2 for examples). The dashed gray line at y = x indicates “no movement.” The black line shows a linear fit applied to the pooled data of all animals, which is given by y = 10.72 + 0.57x, R = 0.97, p < 0.0001 (versus slope = 0). Extensions starting at more extended angles (to the left of the x axis) reached more extended maximal angles, but their amplitude (offset from dashed line) was smaller. (B) The peak velocity of active tibial extensions driven by single FETi spikes increased systematically as the starting angle became more flexed. Values are mean peak velocity (±SD). Each animal is represented by a different symbol (n = 5–9 trials per data point). In three animals, active twitches were elicited from both extended (red) and flexed (black) angles. In a further four animals, active twitches were elicited only from extended angles (red). See also Figure S2.

FETi-driven extensions starting from extended angles had smaller amplitudes and were slower than movements starting from flexed angles (Figures 2 and S2). Full tibial extension (18.6° ± 1.4°, N = 7) was not reached by extension movements driven by single FETi spikes in any locust, regardless of the starting position (Figure 2A). For all locusts, it was therefore possible to find a starting angle (generally near 25°) at which stimulation of FETi with single pulses failed to overcome the counterbalancing passive flexion forces and therefore did not generate a tibial extension.

For FT angles ranging between 30° and 170°, the peak velocity of extension movements varied 26-fold (Figure 2B). Active extensions starting from near the resting range (between 80° and 100°) had velocities between −750°/s and −500°/s. Between-animal and within-animal variability were both small for active movements.

### Passive Movements Can Be Faster Than Active Movements

**Figure 3:**
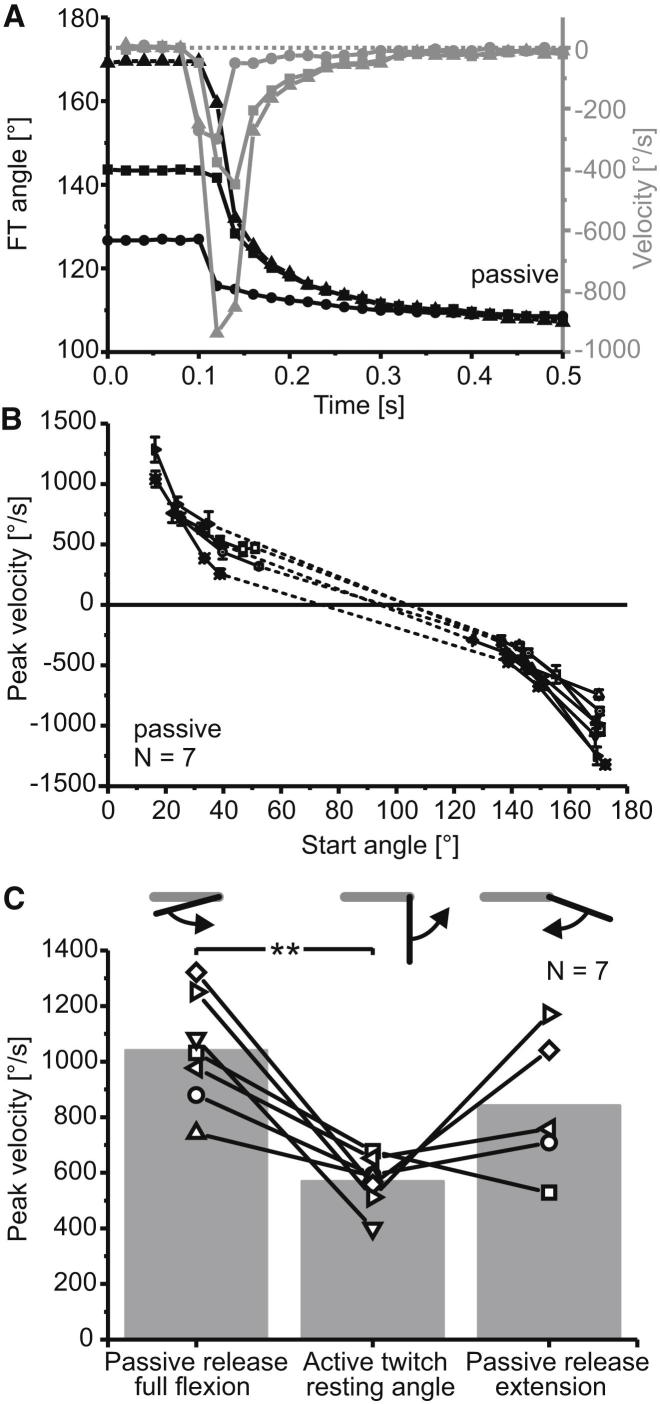
Passive Movements of the Locust Femur Tibia Joint Can Be Faster Than Active, FETi-Driven Movements (A) Passive extensions from three different flexed starting positions. Movements are shown in black; velocities are shown in gray. Upon release (at 0.1 s), the tibia extended toward the resting position. Each curve describes a single trial, and comparable symbols indicate the movement and velocity of the same trial. The dashed line indicates a velocity of 0°/s. (B) Velocities of passive movements differed markedly for different starting angles. Values are mean (±SD) peak velocities of passive tibial movements from seven animals. Positive velocities refer to flexions; negative velocities refer to extensions. The dotted lines connect the extension and flexion data sets for each individual animal. Within this central part of the joint angle range near the normal resting position, the tibia did not move when it was released. (C) Passive extensions or flexions starting from extreme angles were, with one exception, faster than active, FETi-driven extensions starting at the central resting angle. The gray bars show the mean velocity over seven animals, and the symbols joined by black lines show the individual mean peak velocities for each animal separately. Passive extensions from full flexion were significantly faster than active extensions driven by single FETi spikes starting from the resting angle. There was no significant difference between the velocity of active extensions from the resting angle and passive flexions starting from extended angles. Insets show the starting position of the tibia (black) relative to the femur (gray) and the direction of resulting movement (curved arrows). See also Figure S3.

To determine the role of passive forces in generating this angle-dependent modification of the FETi spike-to-movement transfer, we measured entirely passive movements starting from different joint angles. When the tibia of an isolated or denervated locust hind leg was moved away from its resting position and then released, it returned in the absence of motor activity (Figure 3A; see also [8]). Passive extensions from different flexed starting positions all followed the same pattern (Figure 3A). The tibia extended rapidly and then slowed down as it approached the resting position (Figure 3A, black). Release from more flexed positions resulted in faster movements (Figure 3A, gray). Passive extensions usually continued at very low velocities for several seconds.

In all locusts, there was a “resting-state range” in the midrange of joint movement, within which the amplitudes and velocities of passive tibial movements were small and corresponding velocities were not measured (dotted lines in Figure 3B). The mean peak velocity of passive extensions starting from full flexion was 1,040°/s, and that of active extensions starting from the resting angle (where passive forces are negligible) was 570°/s (Figure 3C). Passive extensions from full flexion were therefore 1.8 times faster than active extensions starting from the resting angle (ranging from 1.3 to 2.7 times faster in different animals: t = 5.02, p = 0.002, paired t test, N = 7; Figure 3C). Passive flexions from extended angles were 1.5 times faster than active extensions starting from rest, although this difference was not significant (t = 1.68, p = 0.17, paired t test, N = 5; Figure 3C). This was primarily because some of the movements started from angles that were up to 20° more flexed than full extension and were therefore relatively slow. We took this conservative approach to avoid overextending the tibia. These data demonstrate that passive movements are generally as fast as, and can even be faster than, active movements driven by FETi, the fastest motor neuron driving the strongest and largest muscle of the FT joint.

We determined the contribution of an FETi-driven contraction to overall (active plus passive) tibial movement velocity by subtracting the velocity of passive movements from the velocity of FETi-driven movements. The contribution of an FETi-driven contraction was relatively constant at approximately −550°/s for movements starting over a wide range of joint angles (50° to 140°; see Figure S3). Assisting and resisting passive forces modulated these movements strongly, so that in an intact leg, the velocity of an FETi-driven extension ranged from approximately 0°/s (for a movement starting near full extension) to approximately −1,500°/s (for a movement starting at full flexion, see Figure S3).

### Forces Driving Passive Flexions Originate in the Joint Itself, Whereas Passive Extensions Are Driven by the Extensor Tibiae Muscle

**Figure 4:**
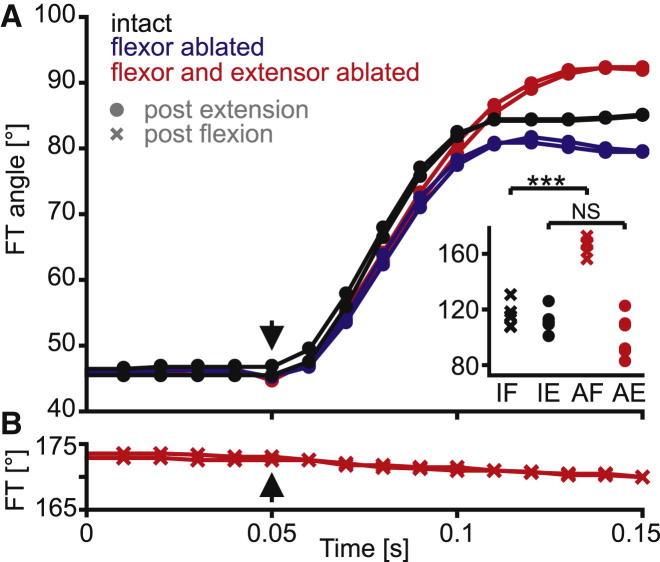
Passive Flexions of a Locust Hind Leg Are Driven by Passive Forces Originating within the Femur-Tibia Joint Itself, Whereas Passive Extensions Are Driven by Passive Forces Arising in the Extensor Tibiae Muscle (A) The time course of passive flexion movements of an intact leg (black symbols), the same leg after flexor tibiae muscle and tendon ablation (blue symbols), and after complete ablation of both flexor and extensor tibiae muscles and their tendons (red symbols). Two trials are shown for each condition. The tibia was released at 0.05 s (arrow). Ablating both muscles had no effect on the velocity of flexion but resulted in a slightly larger total amplitude of movement. The inset shows resting femorotibial joint angles of six intact isolated locust hind legs (black symbols) and the same legs following ablation of both the extensor and flexor tibiae muscles and tendons (red symbols). IF, intact flexed; IE, intact extended; AF, ablated flexed; AE, ablated extended. (B) When both the extensor and flexor tibiae muscles and their tendons were ablated, there was virtually no passive tibial extension following release from full flexion. Data are from the same animal as that shown in (A) and are shown at the same scale (see also Figures 3A and S4). See also Figure S4.

To identify the origin of forces driving passive movements, we compared passive extensions and flexions in intact, denervated locust hind legs with corresponding movements measured after sequential ablation of the flexor and extensor tibiae muscles and tendons (Figure 4).

The initial, fast phase of passive flexions was very similar in intact legs and in both ablated conditions (example movements from one animal are shown in Figure 4A). The resting angle reached following release from full extension was similar in intact legs and after double ablation (compare black and red circles in Figure 4 inset; paired t test: t = 1.54, df = 10, p = 0.15). The relation between starting joint angle and passive flexion velocity was also similar in intact legs and after double ablation (Figure S4 and inset). Passive flexions from extended angles were thus mainly driven by forces arising within the joint itself, with little contribution from passive muscle or tendon forces.

The situation was completely different for passive extensions from flexed joint angles (Figure 4B). When both muscles were ablated, the tibia moved on average only 6.9° ± 5.6° (N = 6). In the animal shown in Figure 4B, these movements occurred at only 42°/s ± 11°/s (n = 5). Following release from full flexion, the final resting angles of doubly ablated legs differed significantly from those of intact legs (compare black and red crosses in Figure 4 inset; paired t test: t = −11.98, df = 10, p < 0.001). In summary, when there were no muscles or tendons attached to the tibia, there was almost no passive tibial extension, but passive flexions were unimpaired.

We progressively dissected the FT joints of locust hind legs to identify the structures driving passive flexions. Even after complete removal of both muscles, their tendons, and all soft cuticular tissues (thus leaving only the two lateral and medial cuticular pivots intact), the tibia still flexed passively, even against gravity.

### Passive Leg Movements in *Pseudoproscopia scabra* and *Carausius morosus*

**Figure 5:**
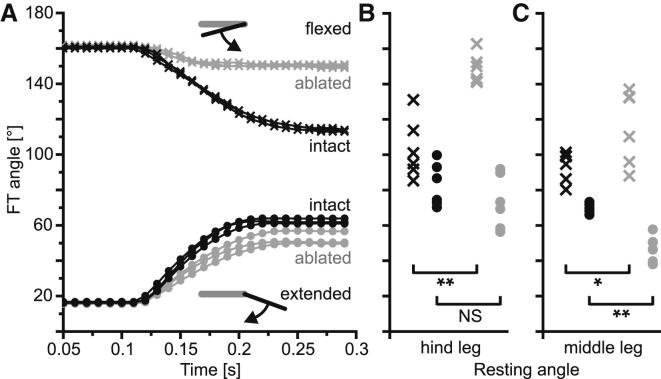
Passive Tibial Movements of Isolated Legs of *Pseudoproscopia scabra* (A) On release from either flexed (crosses) or extended (circles) angles, the tibia of intact hind legs (black curves) moved passively toward a midpoint resting range. Ablation of both the extensor and flexor tibiae muscles and their apodemes (gray curves and symbols) led to a marked reduction in passive extensions (crosses) but had little effect on passive flexions (circles). Insets show the starting position of the tibia (black) relative to the femur (gray) and the direction of resulting movement (curved arrows). (B) The resting angles of hind legs measured 10 s later (symbols and color coding as in A) reflect these differences as described in the Results. Individual legs were always more flexed after flexion than after extension (see Table S2). (C) Corresponding data for middle legs. Symbols and color coding are as in (A). See also Table S2.

In hind legs of the false stick insect *Pseudoproscopia scabra*, as in the locust, release of the tibia from extended or flexed positions was followed by passive movements toward a central resting position (black curves in Figure 5A). Ablation of both the flexor and extensor tibiae muscles had little effect on passive flexion movements from the fully extended position (gray circles in Figure 5A). The tibiae returned to resting positions that did not differ significantly from those of intact legs (Figure 5B, compare black and gray circles; Table S2). In contrast, ablation of both the flexor and extensor tibiae muscles led to slower and much smaller-amplitude passive extensions from the fully flexed position (compare black and gray crosses in Figure 5A). The resting tibial positions of isolated intact hind legs were significantly more extended after passive extension movements than those of legs in which both muscles were ablated (Figure 5B, compare black and gray crosses; Table S2). This shows that in *Pseudoproscopia* hind legs, as in locust hind legs, passive joint forces drive flexions of the tibia, whereas passive muscle forces drive extensions.

The resting angles of isolated intact *Pseudoproscopia* hind legs differed for movements beginning at extended or flexed angles (Figure 5B, compare black crosses and black circles; Table S2), indicating that they have a resting-state range comparable to that of locust hind legs [8].

Tibial resting angles of isolated *Pseudoproscopia* middle legs (Figure 5C; Table S2) followed a similar pattern to that described for isolated hind legs but differed in two important respects. First, ablating both muscles in middle legs had a considerably greater effect on passive flexions than it did in hind legs (the mean difference between black and gray circles in Figure 5C is significantly greater than that in Figure 5B; t = 2.96, p = 0.04, N = 6). Second, ablating both muscles had a smaller effect on passive extensions than it did in hind legs (the mean difference between the black and the gray crosses in Figure 5C is smaller than that in Figure 5B), although this difference was not significant (t = −1.54, p = 0.18, N = 6). These data indicate that, in the isolated middle legs of *Pseudoproscopia*, there is a more symmetrical contribution of passive muscle forces from the extensor and flexor tibiae muscles, and of passive joint forces, than there is in the hind legs of both *Pseudoproscopia* and locust. In particular, the influence of the passive flexor muscle is significantly larger in middle legs than in hind legs. The contribution of passive joint forces to passive flexions is thus smaller in middle legs than in hind legs. In hind legs, the extensor tibiae muscle weighed 51.5 ± 5.6 mg and the flexor tibiae 23.2 ± 2.8 mg, with a mean extensor:flexor ratio of 2.2 ± 0.3 (N = 3, n = 4). In middle legs, the extensor tibiae muscle weighed 2.5 ± 0.7 mg and the flexor tibiae 6.6 ± 1.2 mg, with a mean extensor:flexor ratio of 0.39 ± 0.11 (N = 3, n = 6).

We examined published muscle ablation data for the middle legs of a nonjumping stick insect, *Carausius morosus* (Figure 2 in [7]). In this species, the flexor tibiae muscle is approximately three times stronger than the extensor tibiae muscle [30], and here passive joint forces drive *extensions*, whereas passive forces originating in the flexor muscle drive passive flexions.

In summary, our analyses show that (1) passive joint forces support the weaker flexor muscle in locust and *Pseudoproscopia* hind legs, (2) joint forces are more symmetric in *Pseudoproscopia* middle legs where the extensor muscle is weaker than the flexor muscle, and (3) passive joint forces support the weaker extensor muscle in stick insect legs. Thus, passive joint forces are adapted to the individual strengths of antagonistic muscles in different leg pairs.

## Discussion

### A Concept for the Generation of Cyclic Movements in Insect Limbs

**Figure 6:**
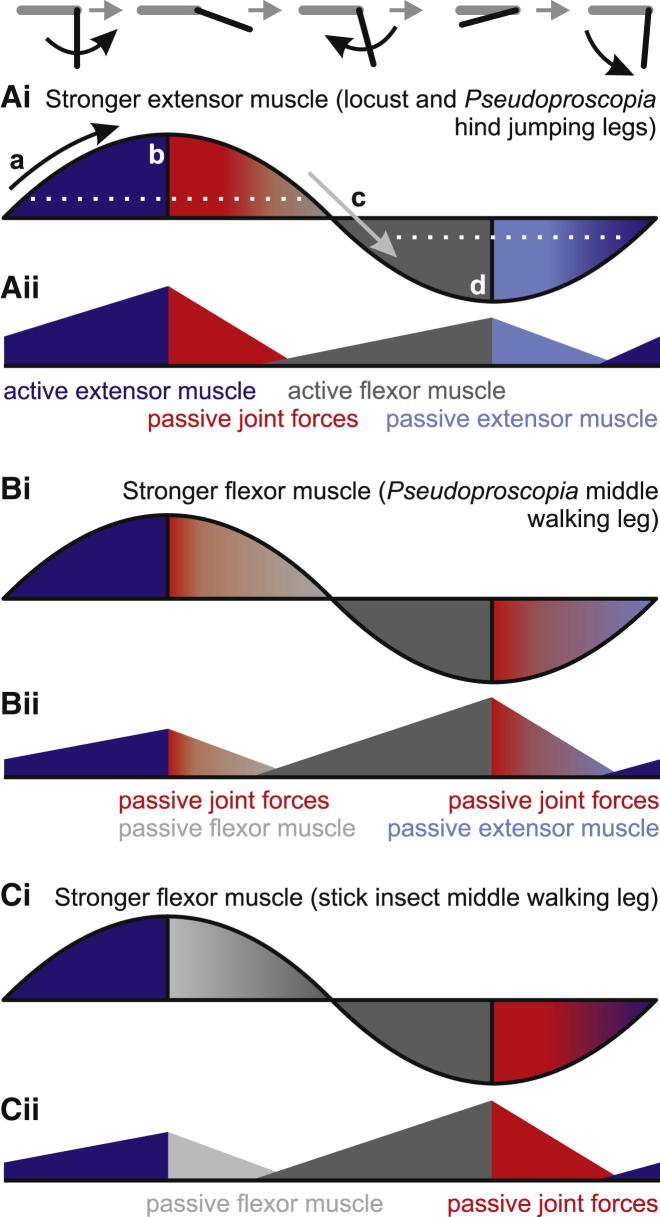
Passive Joint Forces Are Integral to the Generation of Cyclic Limb Movements in Insects The top panel shows the femur (gray) and tibia (black) in different phases of a movement cycle. Curved arrows show direction of tibial movement. (Ai and Aii) Passive joint forces support flexions in the jumping legs of locust and *Pseudoproscopia*. (Ai) FT joint angles during a cyclic extension and flexion movement. The resting range is indicated by white dotted lines. Movements away from the resting range are driven by either extensor tibiae (dark blue, arrow a) or flexor tibiae muscle (gray, arrow c) contractions. In contrast, return movements toward the resting range can be driven by passive joint forces (red) or by passive extensor muscle forces (light blue). The extent to which the tibia can be passively moved toward the midpoint depends on the starting position of the passive return movement and on residual forces of the flexor tibiae and extensor tibiae muscles (regions of overlap between red/gray and dark/light blue). (Aii) Schematic of the changing forces produced by the muscles or joint at different joint angles. The schematic is not intended to characterize precisely the joint-angle or time dependencies of the forces, which are almost certainly not linear as illustrated. (Bi and Bii) In *Pseudoproscopia* middle legs, the flexor muscle is stronger than the extensor, and passive joint forces contribute to both passive flexions and passive extensions (red). Colors and conventions are as in (A). (Ci and Cii) In stick insect middle legs, the relative sizes and strengths of the extensor and flexor tibiae muscles are reversed in comparison to (A). Here, passive joint forces drive tibial *extensions* at flexed joint angles (red), whereas passive flexor muscle forces drive tibial flexions at extended joint angles (light gray). Colors and conventions are as in (A). In behaving animals, passive movements may be assisted by muscle contractions to enhance movement velocities, but they can occur entirely passively, without muscle contractions.

Passive tibial movements starting from extremely flexed or extended angles are faster than active movements starting from the resting angle. Passive movements starting from less extreme angles are slower, but still within the velocity range of active movements. This suggests that natural movements of the tibia can follow a motor control scheme that is different from the classical idea that extensor tibiae motor neurons drive all extensions, and that flexor tibiae motor neurons drive all flexions—at least for movements that do not involve ground contact. We first describe a scheme based on the locust hind leg (Figure 6A; see also [7]) and then contrast this to other legs (Figures 6B and 6C).

All tibial movements away from a central resting angle must be driven by either extensor or flexor tibiae muscle activity (Figure 6Ai, arrows a and c and dark blue/gray shading). If driving motor activity ceases while the tibia is within the resting-state range (dotted lines in Figure 6Ai), tibial movement stops. Once the tibia is driven by active movements to a position that is outside the current resting-state range of the joint, however, cessation of motor activity will allow the tibia to stop and then passively return toward the resting angle. The passive return velocity will depend on the maximal angle of the preceding active movement. In the schematic example of a cyclical tibial extension and flexion movement shown in Figure 6Ai, the tibia is first extended fully by active contraction of the extensor tibiae muscle (arrow a and dark blue shading) starting from the resting position. If the extensor tibiae motor neurons then simply stop firing (point b in Figure 6Ai), the tibia will move back toward the resting angle until it reaches the current resting state (arrow c in Figure 6Ai). This passive movement from full extension (red shading) is driven in locust hind legs by passive flexion forces arising in the joint itself: neither active nor passive muscle or tendon forces are necessary (Figure 6Aii). This passive movement occurs at a joint angle where the flexor tibiae muscle can exert very little force, due to its unfavorable leverage [31]. The force necessary to extend the tibia beyond the resting range is generated by contraction of the much stronger extensor tibiae muscle, which thus indirectly assists in subsequently flexing the tibia. After reentering the resting-state range (arrow c in Figure 6Ai), the flexor tibiae muscle must be activated (gray shading) to continue the movement to more flexed positions (point d in Figure 6Ai). Once flexed beyond the resting-state range, cessation of flexor muscle activity will permit the tibia to move back toward the resting angle under the influence of passive forces originating in the extensor tibiae muscle (light blue shading).

The hind legs of *Pseudoproscopia,* which are specialized for jumping, follow the same scheme as for the hind legs of locust (Figures 6Ai and Aii), but the nonspecialized middle legs of *Pseudoproscopia* are controlled differently. Here, the flexor muscle is stronger than the extensor tibiae muscle, and our data show that passive joint forces contribute to both flexion and extension of the tibia (Figures 6Bi and 6Bii). The situation differs again in the middle leg of the stick insect, where the flexor muscle is also stronger than the extensor (Figure 6Ci), and passive joint forces play a major role in the generation of extensions but not flexions of the tibia (Figure 6Cii).

### Passive Forces Explain Observed Patterns of Motor Activity

Our neuromechanical modeling of cyclical scratching movements in the locust [3] predicted that rates of SETi firing (and thus extensor force production) should be low at the start of extension movements and increase as the tibia extends. This prediction derived from the model’s demonstration that passive extension forces should exceed active ones at the start of the observed movements. Our new data confirm and extend this prediction for both extension and flexion forces. In the model, passive forces arose from spring-like properties of both the extensor and flexor muscles. We now show that, although this is correct for passive extension forces, it is not true for passive flexion forces, which arise in the joint itself. This is important, because muscle forces are transmitted to the tibia through the apodemes (tendons), with the result that the torque they apply varies in a complex way as the effective lever arms of the muscle attachments change with FT angle. Our analysis of the spike-to-movement transfer function of FETi clearly shows that joint-angle-dependent variations in extensor muscle force-length properties [16] must be countered by systematic changes in the extensor muscle lever arm [31] to yield an almost linear transfer function across most of the working range of the joint. Flexor muscle leverage is particularly disadvantageous at extended angles [31], so reliance on passive muscle forces would be relatively ineffective. In contrast, passive flexion forces arising at the joint itself act directly on the tibia. The demonstrated change in strength with joint angle depends not on apodeme lever arms but on the fine structure of the joint.

The prediction that motor activity should be low at the turning point of a cyclical movement [3] becomes intuitively understandable in the light of our data and the motor control scheme proposed in Figure 6 (particularly arrow c in Figure 6Ai). For the light limbs of insects, there is little inertia to be overcome at the start of movement. Even for movements starting at rest (arrow a in Figure 6Ai), motor activity might be expected to begin at relatively low levels and increase as the resisting passive forces also increase. Our data therefore also explain the timing of FETi activity during natural scratching in locusts [5] and during running in the cockroach [32]. Since the same pattern of firing late in extension occurs in both loaded (cockroach) and unloaded (locust) limbs, we suggest that the need to overcome passive flexion forces—some of which originate in the joint itself—is perhaps as important as the need to counteract loading.

An important goal of recent robotics research has been to implement elastic joint actuation by using sophisticated control mechanisms [33], by incorporating physical elastic elements in joint construction [34], or by using biologically inspired control mechanisms that mimic muscle physiology [35]. We show here that elastic structures are indeed present within insect joints and suggest that it might be beneficial to tune the passive properties of artificial limb joints to limb use—particularly where functional requirements dictate asymmetrical actuator characteristics.

### Role of Passive Forces in Natural Behaviors

We show here that passive forces, some intrinsic to joints themselves, make important contributions to slow cyclical movements. We have demonstrated experimentally that passive joint forces can act in ranges of joint angles where neither active nor passive muscle forces would be effective in generating joint torque. The contributions of passive joint forces, and in particular the consequences of this decoupling from muscle moment arms, have been overlooked in most models of limb joint function, with the exception of [36]. Passive joint forces are likely to be unaffected by the patterns or history of limb muscle activation, or by neuromodulators. The relative contributions of different active and passive forces to movements as depicted in Figure 6 will nonetheless differ in different situations. For example, neuromodulation or slow motor activity may induce long-lasting tonus (“catch” [37]) in muscles, or motor inhibition may speed up muscle relaxation [38].

The median peak tibial velocity during aimed scratches of a locust is 590°/s, with an interquartile range of 538°/s (N = 8, n = 151 scratches; D. Calas, personal communication), which is slower than most of the passive movements that we recorded. The mean angular velocity of the hind leg tibia during walking is 200°/s–900°/s (based on [29]), which means that entirely passive movements of the tibia would not be rate limiting during either cyclic aimed scratching or walking. Indeed, we have previously shown (Figure 5 in [5]) that in natural scratching movements, successive tibial extensions driven either entirely passively or by SETi spikes do not differ noticeably from one another.

When a locust kicks [39, 40], the tibia reaches peak extension velocities of typically 46,000°/s. Almost nothing is known about how the large forces involved are dissipated so that the tibia is decelerated without damage to the limb when the kick misses the target. The only mechanism described so far is a plane of weakness in the tibia that absorbs a small part of the energy remaining at the end of a kick [39–41]. Inspection of published tibial movement traces [40] indicates that the tibia decelerates before the joint reaches maximal extension. The passive joint forces that we have described will contribute to this deceleration and thus help protect the FT joint from damage during kicking.

### Passive Forces Show Evolutionary Adaptation

We have demonstrated that in the locust hind leg, which is specialized for jumping, passive muscle forces drive extension movements from flexed angles, whereas passive joint forces drive flexions of the tibia from extended angles. Passive joint forces flexing the tibia are presumably advantageous in the locust leg, since the extensor tibiae muscle is much stronger than the flexor [25], leading to large residual extensor muscle forces which the weaker flexor tibiae needs to overcome during flexions following extensions. The passive joint forces driving flexions represent a functionally important energy transfer from the more powerful extensor to the weaker flexor. The legs of stick insects and cockroaches are adapted to their walking and running lifestyles, and not to jumping. In both cases, the flexor tibiae muscle is stronger than the extensor tibiae (stick insect [30]; cockroach [42]), and in the stick insect, passive joint forces resist flexions, not extensions [7]. These observations suggested to us that passive joint forces might be adapted to different limb functions and insect lifestyles. To test this hypothesis, we examined the role of joint forces in hind and middle legs of a fourth insect, the false stick insect *Pseudoproscopia scabra*. Although this proscopiid is closely related to grasshoppers and locusts, it morphologically and behaviorally resembles stick insects. For example, proscopiids show twig mimesis underpinned by catalepsy that has evolved separately from that in the true stick insects [43]. Proscopiids also retain the locust-like ability to jump and kick using their relatively large hind legs, and these movements are driven by locust-like patterns of muscle activation [44]. In the hind legs of the females used in our study, the extensor tibiae muscle was 2.2-fold heavier than the flexor muscle and presumably generated correspondingly greater forces. We show that in these legs, passive joint forces indeed resist extensions, as predicted by our hypothesis, and presumably contribute to deceleration of the tibia (see e.g. Figure 3 in [43]) at the end of rapid kicks. In *Pseudoproscopia* middle legs, however, the extensor tibiae muscle is lighter than the flexor, and we show that here, joint forces act approximately symmetrically to generate passive extensions and flexions. In particular, the contribution of the passive flexor tibiae muscle is considerably larger in *Pseudoproscopia* than it is in *Schistocerca*. This is again consistent with our hypothesis. We show that similar movements can be generated in different limbs by different patterns of driving forces. Our analyses therefore provide clear evidence that in limbs specialized for different functions in three insect species and four different limbs, passive forces originating in the femur tibia joint itself are tuned to the relative strengths of the antagonist muscles acting at the same joint and are therefore likely to have been shaped by natural selection acting on motor control systems.

## Experimental Procedures

The methods followed those in [8]. In brief, intact adult desert locusts (*Schistocerca gregaria*; Figure S1) were taken from a crowded culture at the University of Leicester maintained under a 12:12 hr light/dark cycle with 36°C daytime and 25°C nighttime temperatures. Experiments were conducted at 23°C–25°C.

### Motion Capture and FETi Stimulation

Locusts were fixed on a platform, the right hind leg was denervated, and all joints proximal to the FT joint were fixed. Fast extensor tibiae (FETi) motor spikes were elicited in the extensor tibiae muscle (Figure S1Aii) using electromyogram wires, and the timing was marked by an LED pulse visible in the video recording. Movements were recorded at 50 Hz [8]. Movement data for Figures 4 and S4 were captured using StreamPix software (NorPix) and a digital video camera (Basler A602fc) recording images of 656 × 490 pixels at 100 Hz. Movements were tracked and joint angle data were computed as described in [26]. The femur-tibia (FT) angle is always given as the external angle, with a straight line projected beyond the femur defined as 0° (see Figure 1 inset). During natural aimed scratching, FETi fires 0–9 spikes per cycle of movement (generally 1–2 spikes per cycle) at firing rates ranging from approximately 3.5 spikes per second (when there is a single spike per cycle) to 36 spikes per second [5]. We therefore stimulated FETi either with individual pulses or with five pulses at 7.5 Hz or 20 Hz. The stimuli were repeated 3–20 times with an intertrial interval of at least 30 s. Movements were generated from FT joint starting positions ranging from fully flexed (∼170°) to fully extended (∼20°). Where movements were elicited at joint angles other than the resting angle, the leg was held in the starting position for 10–30 s by a stiff vertical wire attached to a speaker coil, which then released the tibia 6.7 ms before FETi stimulation.

### Passive Movements in *Pseudoproscopia*

*Pseudoproscopia scabra* (Figure S1B) were obtained from a colony at the University of Cambridge, maintained at room temperature, and fed on bramble leaves. Hind and middle legs were removed from three females (137 ± 15 mm body length, 2.1 ± 0.3 g weight) at the level of the thorax-coxa joint and fixed so that the tibia could move freely in the horizontal plane. Passive movements started from the extreme positions, where the legs were held for 1 s before release. The resting joint angle was measured 10 s after the tibia was released from the fully flexed or extended position. Passive movements were filmed and analyzed using the 100 Hz camera system described above. In three further females (133 ± 10 mm body length, 3.3 ± 1.2 g weight), middle and hind legs were removed, and the extensor and flexor tibiae muscles together with their tendons were dissected out in saline. The individual muscles were dabbed dry on tissue paper and weighed.

### Data Analysis

N refers to the number of animals, and n to the number of trials or legs. Values are given as mean ± SD. Velocities were calculated as a mean over three consecutive video frames. Negative velocities correspond to extensions, and positive velocities to flexions of the tibia. Where appropriate, we interpret faster tibial movements as evidence for higher driving forces at the point of action, which is a reasonable assumption for the open-loop movements (lacking neuronal feedback and ground contact) that we analyzed. We analyzed 2,600 movements of 24 locusts in the course of this study using MATLAB (MathWorks), OriginPro (OriginLab), and custom-written software.
