# Supplementary material for: Passive Joint Forces Are Tuned to Limb Use in Insects and Drive Movements without Motor Activity
Source: Curr Biol. 2013 Aug 5;23(15):1418–26. doi: 10.1016/j.cub.2013.06.024 (PMC3739007; doi:10.1016/j.cub.2013.06.024)
Supplement: Document S1. Figures S1–S4 and Tables S1 and S2 [file mmc1.pdf]

Current Biology, Volume *23*

Supplemental Information

Passive Joint Forces Are Tuned to Limb Use in Insects and Drive Movements without Motor Activity

Jan M. Ache and Thomas Matheson

Author Contributions

J.M.A. designed and carried out the experiments, analyzed the data and wrote the manuscript as part of his Masters Degree at the University of Cologne. T.M. conceived and supervised the overall research program, designed and carried out some experiments and revised the manuscript.

Inventory of Supplementary Information

Figure S1 relates to Figure 1. It illustrates the species used in the study and provides a schematic representation of the relative muscle sizes.

Figure S2 relates to Figure 2. It provides example time course data as used to generate the graphs in Figure 2.

Figure S3 relates to Figure 3. It illustrates the subtraction of passive force curves derived from data plotted in Figure 3B from the summed active + passive force curves plotted in Figure 2B.

Figure S4 relates to Figure 4. It provides quantification of passive flexion velocities that are illustrated as time courses in Figure 4A, and shows that the effects are consistent across animals and the joint angular work range.

Table S1 relates to Figure 1. It provides quantification of the time course data illustrated in the figure.

Table S2 relates to Figure 5. It provides statistical comparisons of contrasts shown in the figure and described in the text.

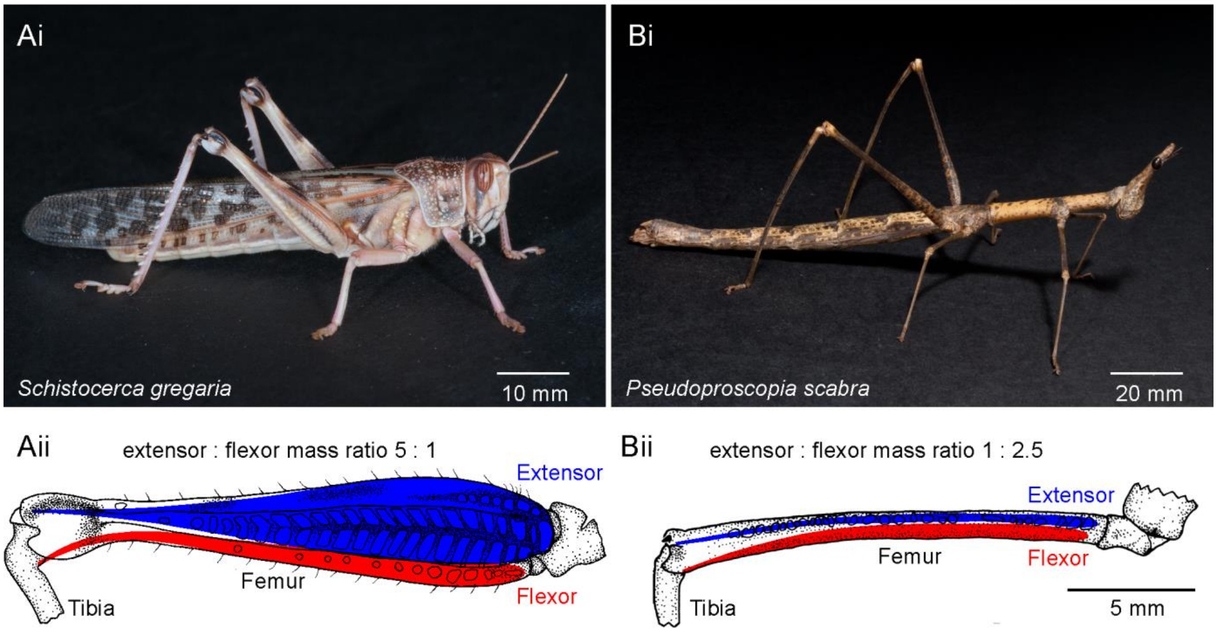

Figure S1. Locusts and False Stick Insects Have Different Leg Morphology, Related to Figure 1

(Ai) Locust *Schistocerca gregaria* showing large hind legs specialised for jumping.

(Aii) Schematic representation of relative extensor tibiae (blue) and flexor tibiae (red) muscle sizes in the hind leg of the locust.

(Bi) False stick insect *Pseudoproscopia scabra* showing large slender hind legs specialised for jumping and relatively unspecialised fore- and middle legs.

(Bii) Schematic representation of relative extensor tibiae (blue) and flexor tibiae (red) muscle sizes in the middle leg of *Pseudoproscopia*. The scale for both schematics is the same. Muscle representations are not intended to reflect details of muscle anatomy.

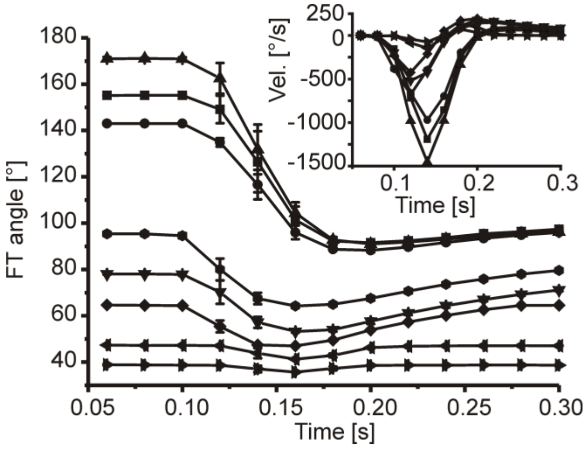

Figure S2. Passive and Active Forces Interact in a Predictable Way in Intact Locust Joints, Related to Figure 2

FETi-driven extensions starting from different FT joint angles in one animal. The inset shows one representative raw velocity curve for each starting angle. Symbols in the inset correspond to those in the body of the figure. The maximally extended position was 21° in this animal, and was not reached in any of the trials. In these experiments, extended starting positions (<80°) were imposed in such a way that the tibia could not return passively to angles more flexed than the starting angle. Passive tibial flexions were therefore not analyzed in this data set. Error bars indicate the SD.

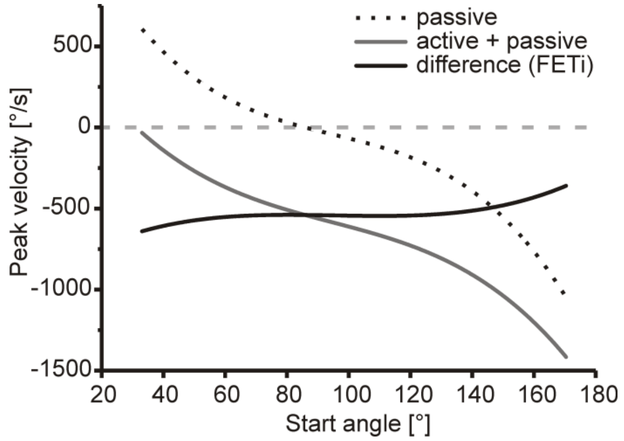

Figure S3. The Interaction of Active and Passive Forces in the Locust Hind Leg, Related to Figure 3

The interaction of active and passive forces was investigated by comparing active and passive movement velocities across the full range of joint angles using cubic fits applied to the mean movement velocities of the 7 animals shown in Figures 2B and 3B. Subtraction of the passive fit from the active fit yielded an estimate of the tibial movement velocity added by a single FETi spike at different joint angles across the work range of the FT joint. The black dotted curve is a cubic fit applied to the pooled data of all passive movements shown in Figure 3B, and the solid grey line is a cubic fit to the pooled active movements shown in Figure 2B. The solid black line shows the difference between these two relationships, and thus represents the velocity added to passive movements by a single FETi spike at different FT joint angles. At extended angles smaller than 40°, passive flexions were faster than active extensions. Despite this, the tibia still extended in response to an FETi spike. The opposite effect was observed for the most flexed angles, where FETi contributed less than in the resting range. N = 7. Only 20% of the difference in FETi contribution to tibial movement velocity occurred in the mid-range between 50° and 140°. Using this method, the velocity of active extensions starting from within the resting range (80°-100°) was estimated to be approximately -550°s^{-1}, which corresponds very well with directly measured values (cf. Figure 2B).

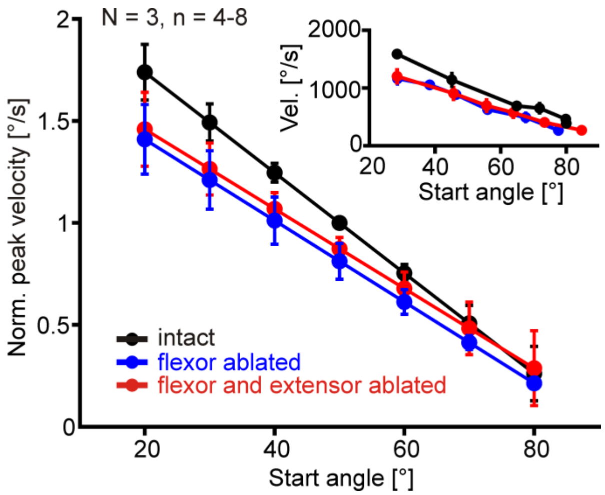

Figure S4. Averaged Passive Tibial Flexion Velocities for Three Locusts, Related to Figure 4

To pool the data, a linear fit was applied to the raw data (e.g. inset) of the intact condition for each animal separately. The velocities of all movements made under all conditions for each animal were then normalized to the velocity at 50° FT angle yielded by the linear fit to that animal's 'intact' data. The normalized data were then pooled across all 3 animals for 7 joint angles within the range covered by the original trials and fitted linearly. Starting FT joint angle and passive movement velocity were strongly correlated in all animals and under all conditions, with all $R > 0.95$ (except animal 1, intact $R > 0.92$) and all $p < 0.01$ (except animal 1, intact $p < 0.03$). Passive flexions of intact legs were slightly faster than those of legs in which the flexor tibiae or both muscles had been ablated. The within-animal variability of passive flexion velocities was small in all three conditions (standard deviation bars in the inset).

Table S1. Comparison of Active Tibial Extension Movements Driven by FETi Stimulation with Subsequent Passive Return Flexion Velocities, Related to Figure 1

	Peak velocity of active extension [°/s]	Peak velocity of passive flexion [°/s]	Maximally extended angle [°]	n
Single FETi spikes	810 ± 90	160 ± 6	64.2 ± 0.6	9
5 FETi spikes, 7.5 Hz	850 ± 26	254 ± 8	25.2 ± 0.3	8
5 FETi spikes, 20 Hz	860 ± 33	177 ± 19	20.8 ± 0.3	9

The maximally extended angles were reached at the end of FETi stimulation, and therefore indicate the effective starting angle for the passive return flexion movement. These data are for one representative example animal of 7 analysed. Data were not compared across animals as there was considerable between-animal variability of start angles and thus peak extension angles, which itself leads to markedly different passive movements, even in the absence of prior muscle contraction (see Figure S2, Figure 3A, main text).

Table S2. Statistical Comparisons of Muscle Ablation Effects in *Pseudoproscopia scabra* Hind and Middle Legs, Related to Figure 5

Leg, comparison	Significance	t	p	df
Hind leg, intact flexed vs. intact extended	***	6.94	4.7×10^{-4}	5
Hind leg, intact flexed vs. ablated flexed	**	5.88	0.001	5
Hind leg, intact extended vs. ablated extended	NS	-1.37	0.11	5
Hind leg, ablated flexed vs. ablated extended	***	-8.22	2.2×10^{-4}	5
Middle leg, intact flexed vs. intact extended	**	-6.79	5.3×10^{-4}	5
Middle leg, intact flexed vs. ablated flexed	*	3.78	0.0064	5
Middle leg, intact extended vs. ablated extended	**	-6.24	7.7×10^{-4}	5
Middle leg, ablated flexed vs. ablated extended	***	-9.98	8.6×10^{-5}	5

Flexed, passive release from full flexion; extended, passive release from full extension; ablated, after ablation of both the extensor and flexor tibiae muscles. Data were tested for statistical significance using one-sided t-tests with Bonferroni correction for multiple comparisons. The Bonferroni corrected p-values used to assign significance were 0.0125 (*), 0.0025 (**) and 0.00025 (***). NS, not significant.
